# A patient‐derived organ‐on‐a‐chip platform for predicting chemotherapy response in locally advanced colorectal cancer

**DOI:** 10.1002/btm2.70190

**Published:** 2026-11-02

**Authors:** Hua Zhou, Xinyu Gu, Weilong Chang, Lin Zhou, Ruixuan Cao, Xin Wang, Liuyan Deng, Shengping Xiao, Sen Ye, Xiaohua Qian, Zhiyong Xie, Shipeng Li, Xin Xie, Haiqing Bai

**Affiliations:** ^1^ Endocrinology and Metabolism Center, Henan Key Laboratory of Rare Diseases The First Affiliated Hospital, and College of Clinical Medicine of Henan University of Science and Technology Luoyang Henan China; ^2^ Henan Academy of Innovations in Medical Science Zhengzhou Henan China; ^3^ Henan Key Laboratory of Cancer Epigenetics, Cancer Institute, The First Affiliated Hospital, College of Clinical Medicine, Henan University of Science and Technology Luoyang Henan China; ^4^ Department of Gastrointestinal Surgery the First Affiliated Hospital of Zhengzhou University Zhengzhou China; ^5^ Yaosu Technology Building 10, Caohejing Nanqiao Science and Technology Park, 6055 Jinhai Highway, Fengxian District Shanghai China; ^6^ Xellar Biosystems Boston Massachusetts USA; ^7^ Department of Hepatopancreaticobiliary Surgery Henan Provincial People's Hospital, Zhengzhou University Zhengzhou China

**Keywords:** cancer‐on‐chip, colorectal cancer, drug screening, organ chip, precision oncology

## Abstract

Colorectal cancer (CRC) remains a leading cause of mortality, underscoring the urgent need for predictive precision medicine tools. While patient‐derived organoids have advanced drug screening, they often lack the dynamic physical microenvironment found in vivo. Organ‐on‐a‐chip (OOC) systems offer superior physiological relevance; however, their translation from bench to bedside has been stalled by a lack of validation against clinical patient outcomes. Here, we bridge this critical translational gap by demonstrating the feasibility and predictive utility of a scalable, patient‐derived OOC (PDOC) platform. From a cohort of 14 primary locally advanced CRC cases, we engineered 8 patient‐specific PDOCs that maintained high‐fidelity histological recapitulation of the parental tumor tissue. Leveraging an automated, high‐content imaging workflow alongside metabolic assays, we profiled responses to standard‐of‐care chemotherapies (irinotecan, FOLFOX, and FOLFIRI). The platform successfully stratified patients into distinct phenotypic response groups, revealing specific sensitivities to FOLFOX versus FOLFIRI. Crucially, we present preliminary clinical evidence supporting these ex vivo predictions: in a 7‐month follow‐up, all patients identified as FOLFOX‐sensitive by our platform remained disease‐free post‐surgery. These findings support the PDOC platform not just as a complex in vitro model, but as a viable, predictive technology for functional precision oncology. This work represents an important step toward integrating microphysiological systems into prospective clinical decision‐making for CRC and other solid tumors.

**Translational Impact Statement**

This work augments the current understanding of interpatient drug response heterogeneity in locally advanced colorectal cancer and should provide clinicians with actionable knowledge to guide personalized therapy selection via a scalable patient‐derived organ‐on‐a‐chip platform that rapidly profiles individual tumor sensitivity to diverse chemotherapeutic and targeted regimens, potentially improving treatment outcomes and minimizing unnecessary toxicity.

## 1 INTRODUCTION

Colorectal cancer (CRC) poses a major global health challenge, ranking as the third most common cancer and the second leading cause of cancer‐related deaths worldwide.^1^ Despite progress in early detection and the adoption of combination chemotherapy such as FOLFOX and FOLFIRI, patient outcomes—particularly in cases of locally advanced or metastatic disease—remain highly heterogeneous, associating with substantial toxicity and limited survival benefit.^1^, ^2^ This variability in treatment response underscores a key limitation of current therapeutic strategies and highlights the urgent need for a more predictive approach that can guide optimal regimen selection.

Historically, preclinical cancer research has relied on two‐dimensional (2D) cell lines and animal xenograft models. While these systems have been foundational, they often fail to capture the complex three‐dimensional architecture, cellular diversity, and dynamic tumor microenvironment (TME) of human cancers.^3^, ^4^ As a result, they exhibit limited clinical translatability and contribute to high rates of drug attrition.^5^, ^6^ The advent of patient‐derived organoids (PDOs) represents a major advancement in this field. These self‐organizing, three‐dimensional cultures, established directly from patient tumor tissue, preserve the genetic and phenotypic features of their tumor of origin and have demonstrated clinical utility in predicting therapeutic responses to chemotherapy, targeted therapy, and immunotherapy in a broad range of tumor types,^2^, ^3^, ^7^ including locally advanced or metastatic CRC.^8^, ^9^, ^10^, ^11^, ^12^, ^13^, ^14^, ^15^, ^16^, ^17^, ^18^, ^19^ However, PDOs typically lack key components of the native TME, such as immune cells, fibroblasts, and vasculature, and do not recapitulate physiological cues—such as nutrient gradients and mechanical forces—that critically influence tumor behavior and response to treatment.^20^ PDOs are also limited by high costs, variable success rates, and a lack of standardization, all of which hinder their clinical utility.^4^, ^6^

Microfluidic organ‐on‐a‐chip (OOC) has emerged as a next‐generation technology that may overcome these limitations. By integrating microengineering with advanced cell culture techniques, OOCs can recapitulate human pathophysiology with high fidelity, incorporating three‐dimensional (3D) tissue architecture, oxygen and nutrient gradients, dynamic fluid flow, and tissue–tissue interfaces to more precisely mimic the TME in patients than organoids or other ex vivo cancer models.^4^, ^21^ Despite great promise, the application of OOC technology to human clinical samples has been scarce, due to challenges such as high costs, low throughput, and cumbersome setup.^22^, ^23^ In our previous work, we developed a scalable, cost‐effective OOC platform combined with imaging‐based analysis to quantify drug responses.^24^, ^25^ We demonstrated its feasibility using cancer cell lines; however, whether it could be applied to patient‐derived samples at scale remained undetermined.

In the present study, we addressed this gap by generating patient‐derived organ‐on‐a‐chip (PDOC) models from a cohort of patients with locally advanced CRC. We evaluated drug responses to standard‐of‐care chemotherapy regimens using the conventional adenosine triphosphate (ATP) assay and an automated imaging‐based analysis pipeline and compared the results with clinical responses. This work represents a key step toward clinical use of OOC technology in drug‐testing based precision oncology and paves the way for future clinical trials.

## 2 RESULTS

### 2.1 Culture and characterization of PDOC for CRC

We included 14 patients diagnosed with locally advanced CRC (stage III) who had not received any prior treatment at the time of surgical resection. Resected tumor tissues were minced, enzymatically digested into single cells, and cultured as organoids (Figure 1a), following established protocols.^13^ Organoid cultures were successfully established from 13 of 14 patients, yielding a success rate of 92.9%, which is consistent with previous reports.^16^, ^17^

**FIGURE 1:**
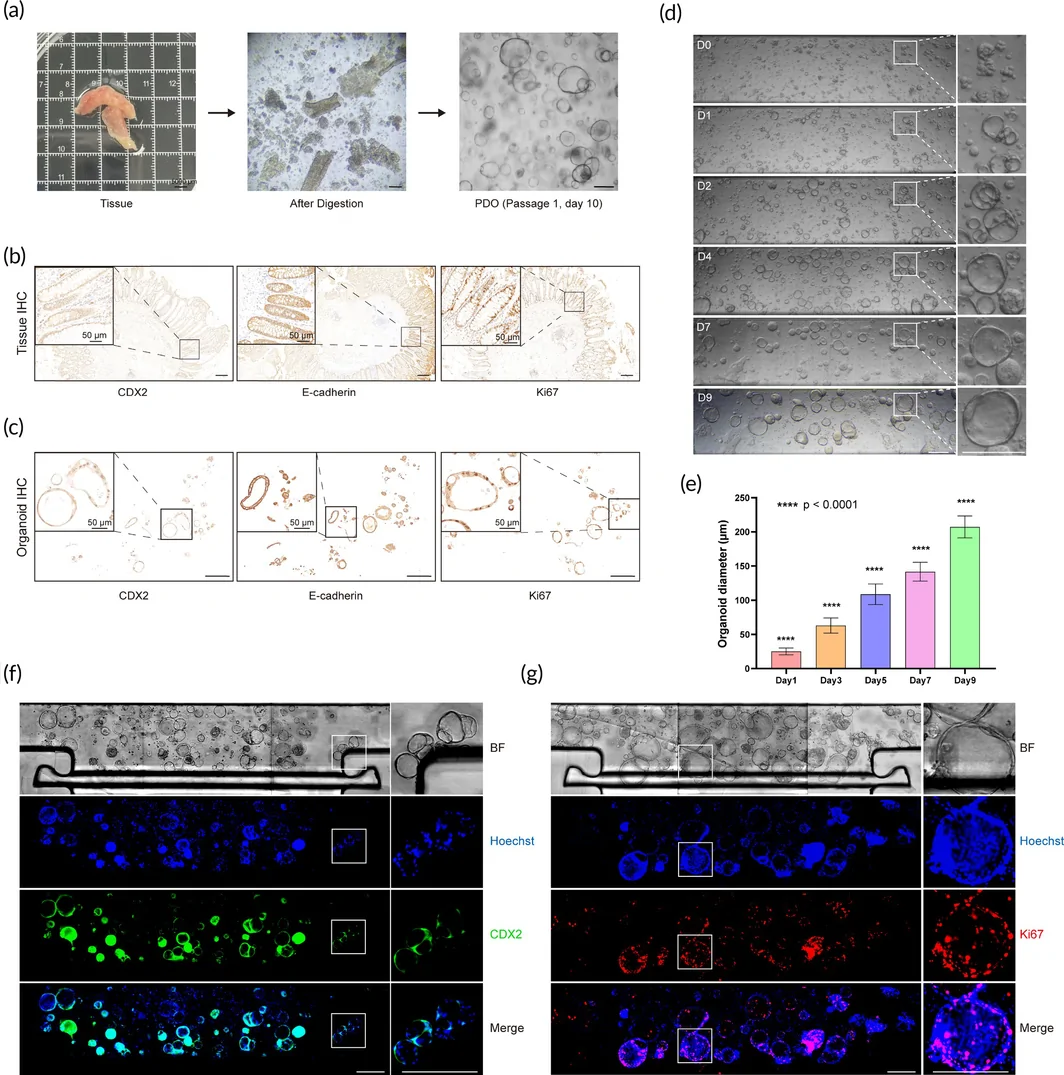
Culture and characterization of PDOC for CRC. (a) Workflow for establishing PDOs from minced and digested CRC surgical tissue (passage 1, day 10). (b, c) Immunohistochemistry for CDX2, E‐cadherin, and Ki‐67 in primary CRC tissue and corresponding PDOs. (d) Representative bright‐field images of PDOC. (e) Organoid growth in the microfluidic chip over time. Organoid diameter was measured on days 1, 3, 5, 7, and 9. Data are shown as mean ± SD (*n* = 10). One‐way analysis of variance (ANOVA) followed by Tukey's multiple comparisons test. ****p < 0.0001 versus day 1. (f, g) Bright‐field and immunofluorescence images showing CDX2 and Ki‐67 expression in PDOC. Scale bars: 200 μm.

Immunohistochemistry confirmed that the organoids retained key tumor marker expression from the original tumors, including CDX2, E‐cadherin, and Ki‐67 (Figure 1b,c). CRC organoid‐derived cells were then seeded into the OC‐Plex microfluidic platform, a novel thermoplastic system featuring dual‐channel fluidics and 8‐unit multiplexing capacity as previously characterized.^24^, ^25^ Bright‐field (BF) imaging revealed robust organoid growth, with organoids reaching a diameter of approximately 200 μm and maintaining viability for at least 9 days (Figure 1d). Quantitative analysis further revealed a time‐dependent increase in organoid size within the chip (Figure 1e, Table S1, Supporting Information). Notably, PDOCs maintained excellent viability and progressive expansion to a maximal diameter of approximately 800 μm after 27 days of continuous culture (Figure S1A). In addition, immunofluorescence analysis further confirmed the sustained expression of CRC tumor markers (CDX2, Ki67) within the chip microenvironment (Figures 1f,g and S1B). Collectively, we established a robust PDOC model that enables long‐term stable culture of CRC organoids.

### 2.2 Establishing an automated imaging analysis pipeline to evaluate drug effect

Low‐cost and reliable quantification of drug response remains a major challenge in functional precision oncology.^6^ Our previous work^24^ has demonstrated that imaging‐based approaches offer greater accuracy and resolution than conventional cell viability assays such as the CellTiter‐Glo (CTG) assay. To further validate this comparison with clinical samples, we developed an automated image analysis pipeline for quantifying drug responses through morphological and viability profiling. In this workflow, CRC organoids were cultured in chips for 4 days followed by 4‐day drug exposure, subsequently stained with live/dead markers (Hoechst/PI), and subjected to high‐content imaging (HCI) to acquire BF and fluorescent images (Figure 2a). For automated imaging analysis, BF images were first used to define a region of interest (ROI) corresponding to the interface region of the device (Figure S2A). Image segmentation masks were then applied to fluorescent images stained with Hoechst and PI to distinguish live (Hoechst‐positive, PI‐negative) and dead (PI‐positive) cells, enabling calculation of cell viability (Figure S2B). Finally, this imaging‐based quantification was validated against the parallel CTG assay to ensure methodological robustness.

**FIGURE 2:**
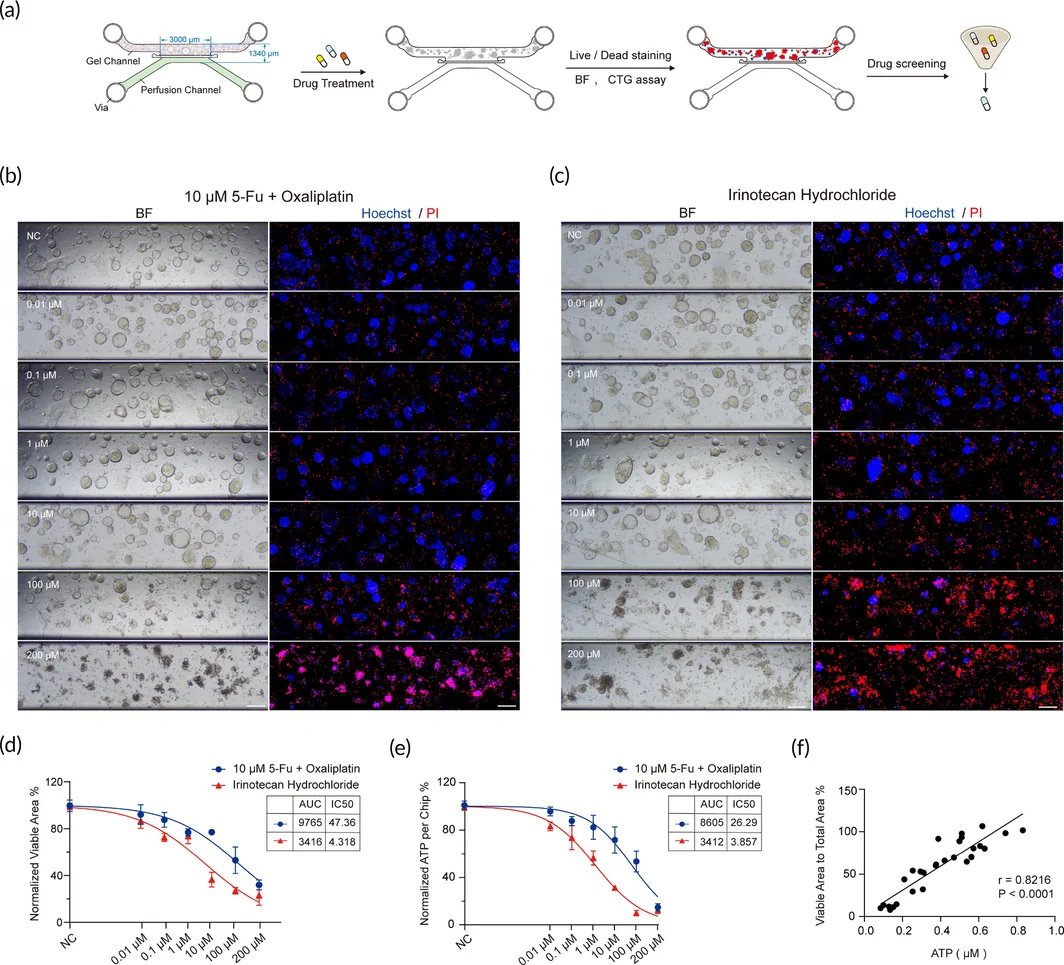
Establishment of an imaging‐based analysis pipeline to evaluate drug effects. (a) Schematic of the workflow for drug testing in PDOC, integrating AI‐assisted imaging analysis with ATP‐based viability assays. (b, c) Representative bright‐field and corresponding Hoechst/PI fluorescence images. PDOCs were treated on day 4 with 10 μM 5‐FU and the indicated concentrations of oxaliplatin (b) or irinotecan hydrochloride (c). Live/dead staining and ATP measurements were performed after 4 days of drug exposure. Scale bar: 200 μm. (d, e) Dose–response curves generated from imaging‐based viability (d) and the ATP assay (e). Data represent mean ± SD; *n* = 3 replicates. (f) Correlation between ATP level and live/dead staining‐based viability measurement.

To evaluate the feasibility of this workflow, we assessed drug responses in organoids derived from a representative patient using two regimens: (i) 5‐Fluorouracil (5‐FU; 10 μM) combined with oxaliplatin, and (ii) irinotecan hydrochloride. Both regimens were tested at six concentrations (0.01, 0.1, 1, 10, 100, and 200 μM). BF and immunofluorescence imaging confirmed significant viability suppression at 200 μM for both regimens (Figure 2b,c). Dose–response curves (Figure 2d,e) demonstrated markedly higher sensitivity to irinotecan hydrochloride than to the 5‐FU + oxaliplatin combination, as reflected by the lower IC_50_ and area under the curve (AUC) values. Furthermore, viability results obtained using the imaging‐based method showed a high level of concordance with CTG assay measurements, with a Pearson correlation coefficient of 0.8216 (Figure 2f). This differential drug response was further confirmed by quantitative viability assessments using both image‐based and ATP‐based analyses (Figure S3A, B) (two‐way analysis of variance (ANOVA), treatment × concentration). Together, these findings validate the robustness and reliability of the imaging‐based workflow for discriminating distinct drug sensitivity profiles.

Collectively, we have established a clinical‐grade drug response profiling workflow for CRC by integrating the OC‐Plex microfluidic platform with HCI phenotyping, enabling robust in vitro assessment of therapeutic efficacy.

### 2.3 Optimization of treatment window for measuring drug response

To determine the optimal treatment duration for capturing drug efficacy in PDOC models, we evaluated the temporal response to the FOLFOX mimic regimen (5‐FU + oxaliplatin). Organoids were cultured for 4 days to allow establishment, followed by drug exposure, and viability was assessed at 3, 4, and 6 days post‐treatment.

Across all time points, the regimen induced a dose‐dependent reduction in viability, as confirmed by BF and immunofluorescence‐based morphological evaluations (Figure 3a–c), as well as image‐based and ATP‐based dose–response curves (Figure 3d–i). This was further corroborated by quantitative imaging and CTG assays (Figure S4A, B) (two‐way ANOVA, time × concentration). Notably, the dose‐dependent effect was already evident at day 3 (Figure 3d), and the response profiles at this early time point were consistent with those observed at days 4 and 6 (Figure 3f,h), indicating that a 3‐day exposure is sufficient to capture the drug's efficacy trend. Given the clinical imperative for rapid turnaround in personalized sensitivity testing, we selected the 3‐day treatment window for all subsequent experiments on the PDOC platform.

**FIGURE 3:**
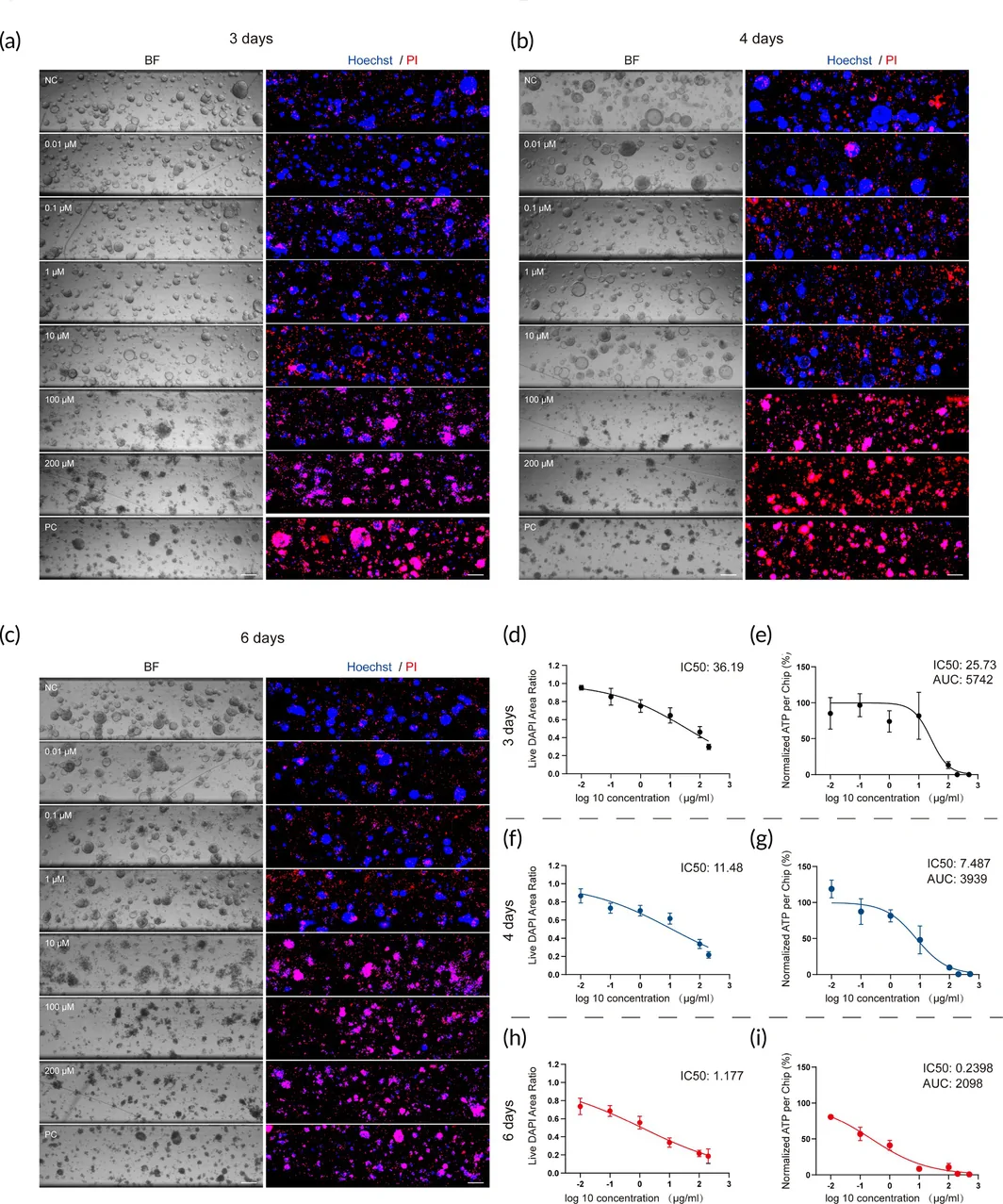
Optimization of the treatment window for drug response measurement. (a–c) Representative BF and live/dead fluorescent images of PDOCs treated with drugs for 3, 4, or 6 days. (d, f, h) Drug dose–response curves generated from live/dead staining. (e, g, i) Corresponding dose–response curves derived from the ATP viability assay. Scale bar: 200 μm. Data are presented as mean ± SD; *n* = 3 replicates.

### 2.4 PDOC recapitulates drug response heterogeneity

PDOC‐based drug testing was performed on 8 of the 13 patients for whom PDOs were successfully established (Table S2). Live/dead imaging analysis revealed heterogeneous responses to treatment across patients. Six patients (P001, P005, P009, P011, P014, and P015) exhibited greater sensitivity to 5‐FU + oxaliplatin (FOLFOX mimic) (Figure 4a, white asterisk), whereas the remaining two (P007, P010) responded more strongly to 5‐FU + irinotecan (FOLFIRI mimic) (Figure 4a, white asterisk). These response patterns were consistent across multiple readouts, including automated imaging‐based viability analysis (Figure 4b) and CTG assay measurements (Figure 4c).

**FIGURE 4:**
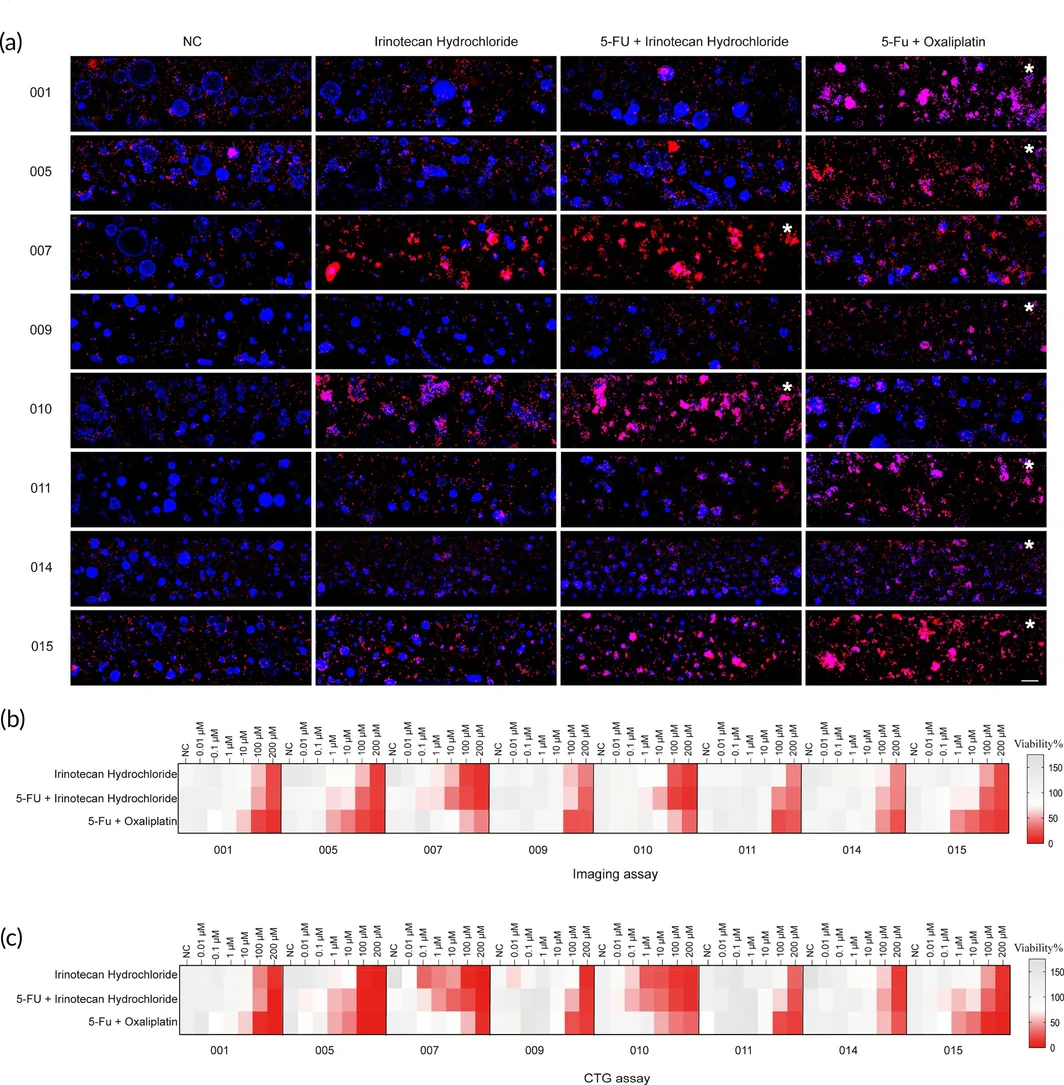
PDOC capturing heterogeneity in patient response to chemotherapy. (a) Representative live/dead fluorescent images of PDOCs following 3 days of drug treatment. PDOCs were treated with 10 μM 5‐FU and the indicated concentrations of irinotecan hydrochloride or oxaliplatin or irinotecan hydrochloride alone. Only data from the 100 μM condition are shown. Scale bar: 200 μm. A white asterisk (*) indicates the treatment eliciting the greatest sensitivity for each individual patient. (b, c) Heatmaps illustrating heterogeneous cell viability profiles across PDOCs derived from eight patients, as quantified by the imaging‐based assay (b) and the ATP assay (c). Data represent mean values from three replicate chips.

To preliminarily assess the predictive potential of the PDOC platform, we performed a retrospective analysis of clinical treatment outcomes. Among patients who received FOLFOX or mFOLFOX6 in clinical care, three (P009, P011, and P014) exhibited concordant sensitivity to the FOLFOX mimic in the PDOC model. In contrast, patients P007 and P010, who were clinically treated with FOLFOX, demonstrated greater sensitivity to FOLFIRI in PDOC assays. Both patients remain progression‐free at the current, albeit short, follow‐up (7 months). These findings provide early evidence supporting the PDOC platform's potential utility in guiding patient‐specific therapeutic strategies for CRC.

## 3 DISCUSSION

This study establishes a scalable PDOC platform that effectively bridges translational gaps in CRC precision oncology. We successfully generated histologically validated models from 13 treatment‐naïve locally advanced CRC patients, demonstrating long‐term culture stability (>27 days) and physiological drug response recapitulation. Our integration of thermoplastic microfluidics with automated HCI enabled high‐resolution profiling of chemotherapy sensitivity hierarchies, revealing distinct patient subgroups responsive to FOLFOX (six patients) versus FOLFIRI (two patients). This stratification capability addresses a critical unmet need in locally advanced CRC management, where current genomic biomarkers fail to predict chemotherapy benefit in over 40% of cases.^26^

Preliminary clinical correlation showing alignment between PDOC‐predicted FOLFOX sensitivity in three patients, while requiring validation in larger cohorts, suggests potential clinical utility. Notably, two patients predicted in vitro to be more sensitive to FOLFIRI exhibited no significant progression after surgery and FOLFOX chemotherapy. This discrepancy may be explained by complete tumor resection or the short follow‐up time. Longer follow‐up is ongoing; should recurrence occur, FOLFIRI may represent an optimal alternative regimen. In summary, the preliminary concordance between PDOC sensitivity and short‐term clinical outcomes is highly encouraging and suggests that OOC technology could eventually guide first‐line therapy selection, potentially sparing patients from the toxicity of ineffective regimens.

A key highlight of our study is the development of a robust, scalable, and analytically advanced platform that addresses several limitations of existing models. While PDOs have proven valuable for drug screening,^9^, ^10^ they often lack the dynamic fluid flow, mechanical cues, and potential for multi‐cellular integration (e.g., endothelial cells, immune cells) that are intrinsic to OOC platforms. Our use of a novel thermoplastic, multiplexed microfluidic device (OC‐Plex) enhances scalability and reduces cost compared to many PDMS‐based chips,^27^, ^28^ mitigating a significant barrier to clinical adoption. Furthermore, we advanced the field of quantitative drug response analysis by establishing a highly concordant, automated imaging workflow that provides rich morphological data alongside viability metrics, surpassing single‐readout endpoint assays such as CTG. This allows for a more nuanced assessment of drug effects, as demonstrated by our ability to distinguish subtle differences in sensitivity between FOLFOX and FOLFIRI regimens.

Despite these advances, our study has limitations including a small cohort size (*n* = 8 profiled) and simplified TME composition lacking immune/stromal components. Future work should incorporate autologous immune cells to evaluate immunotherapies and expand to targeted agents. The early separation of drug response curves at 72 h supports the clinical feasibility of rapid turnaround. Looking forward, prospective validation in multi‐center trials correlating PDOC predictions with RECIST responses will be essential for clinical adoption. This platform represents more than a technological advance—it offers a paradigm shift from static genomic stratification toward functional therapy selection based on dynamic tumor behavior. To our knowledge, this is among the first demonstrations of clinical‐grade CRC‐on‐chip models derived exclusively from treatment‐naïve locally advanced cancers, our work provides a framework for deploying organ‐chip technology in functional precision oncology trials across solid tumors.

## 4 MATERIALS AND METHODS

### 4.1 Human specimens and ethics statement

Primary tumor specimens were obtained from 14 patients diagnosed with locally advanced CRC undergoing surgical resection at the First Affiliated Hospital of Zhengzhou University, following informed consent under protocols approved by the Institutional Review Board (IRB #2025‐KY‐0113‐002). Clinical data, including age, tumor stage, and subsequent therapeutic regimens, were collected from medical records for correlation with PDOC drug response profiles (Table S2). Fresh tumor tissues were collected in cold MACS Tissue Storage Solution (Miltenyi, 130‐100‐008) and processed within 24 h of excision.

### 4.2 Sample processing

Patient‐derived tissues were washed in ice‐cold Dulbecco's phosphate‐buffered saline (DPBS) (supplemented with 1% antibiotic‐antimycotic: penicillin–streptomycin‐amphotericin B) with gentle agitation in 50 mL tubes (3× washes until supernatant clearance). Tissues were transferred to 100 mm culture dishes containing 2 mL DPBS to maintain hydration, with morphological documentation under a stereomicroscope. Following quality assessment, tissues were aseptically dissected into three segments (designated for: culture, cryopreservation, and paraffin embedding). The culture segment was minced into 1–2 mm^3^ fragments, digested in pre‐warmed Tumor Tissue Digestion Solution (Mogengel, 0818L05L), and incubated at 37°C/5% CO_2_ for 20–50 min. Digestion was terminated when >80% tissue dissociation was microscopically confirmed (extended incrementally if required).

The suspension was filtered through 100 μm cell strainers into 50 mL tubes, diluted to 30 mL with DPBS, and centrifuged (300 g, 5 min, 4°C). Optional erythrocyte clearance: Pellet resuspension in 1 mL ACK lysis buffer (Thermo, A1049201) for 2–3 min RT incubation, followed by centrifugation. Final pellets were resuspended in 1 mL DPBS, with 10 μL aliquots mixed 1:1 with 0.4% trypan blue (Thermo, 15250061) for viability assessment using automated cell counters (Countess II, Thermo).

### 4.3 PDO culture

Cell suspensions were resuspended in ice‐cold growth factor‐reduced Matrigel (Corning, 356231) at 4 × 10^5^ cells/mL and maintained on a 4°C cooled surface. Aliquots of 50 μL gel‐cell mixture were seeded into 24‐well cell culture plates. The plate was transferred to a 37°C/5% CO_2_ incubator for 30‐min gel polymerization. Subsequently, 800 μL of Human Colorectal Cancer Organoid culture medium (Mogengel, 807T001) was added to each plate. Medium was replaced every 48–72 h until experimental endpoint.

### 4.4 PDOC culture

OC‐Plex 8 microfluidic devices (Xellar Inc. USA), each containing eight independent chips, were used to generate PDOCs. Each chip consisted of two channels, including a gel channel for cell seeding. A total of 1.5 μL of Matrigel (Corning, 356231) mixed with dissociated PDO cells at a density of 5 × 10^6^ cells/mL was loaded into the gel channel. To prevent gel deformation during polymerization, 50 μL of phosphate‐buffered saline (PBS) was added above the ribbed area. Devices were incubated at 37°C with 5% CO_2_, and after 30 min of gel polymerization, 100 μL of culture medium was added to both the gel and perfusion channel ports. Devices were placed on a shaker for dynamic culture. Culture medium was replenished every 2–3 days until drug treatment.

### 4.5 Immunohistochemistry and immunostaining

Cells were gently washed with PBS to remove residual medium, followed by fixation in 4% paraformaldehyde (PFA, Sigma, 158127) for 15 min at 25°C. After three 5‐min PBS washes, permeabilization was performed using 0.1% Triton X‐100 (Thermo, 28314) for 10 min at 25°C. Non‐specific binding sites were blocked with 5% BSA (Sigma, A7906) or 10% fetal bovine serum (Gibco, 16140071) for 30 min at 25°C. Primary antibodies (CDX2, Servicebio, GB151500; E‐cadherin, Abcam, ab231303; Ki67, Servicebio, GB151142) at 1:500 dilution were applied and incubated overnight at 4°C. Following three 5‐min PBS washes, fluorophore‐conjugated secondary antibodies (FITC conjugated Goat Anti‐Rabbit IgG, Servicebio, GB22303; Cy3 conjugated Goat Anti‐mouse IgG, Servicebio, GB21301) at 1:500 dilution were incubated for 1 h at 25°C protected from light. After final PBS rinses, nuclei were counterstained with 10 μM Hoechst (Thermo, 62249) for 5 min. Chips underwent three additional PBS washes before fluorescence imaging (Molecular Devices ImageXpress Micro Confocal).

### 4.6 Drug treatment

The compounds used in these studies, 5‐Fu (KKL Med, KM17926), Irinotecan hydrochloride (KKL Med, KM9965), were individually prepared in sterile DMSO as 1000× stocks (0.01, 0.1, 1, 10, 100, and 200 mM), aliquoted, and stored at −80°C. On the day of dosing, each compound was diluted with growth medium at a ratio of 1:1000 to achieve final concentrations of 0.01, 0.1, 1, 10, 100 and 200 μM. Vehicle control was prepared by diluting 100% sterile DMSO to 0.1%. For oxaliplatin (KKL Med, KM16240), the drugs were prepared as 2 mM master mixes in sterile water and diluted in growth medium to final concentrations of 0.01, 0.1, 1, 10, 100, and 200 μM, respectively, before use. Medium was removed completely from each OC‐Plex chip and replaced with medium containing compound or vehicle for 72–144 h prior to endpoint viability assessment.

### 4.7 Viability staining

Viability staining solution was prepared by diluting Hoechst 33342 (Thermo, 62249) 1:2000 to a final concentration of 10 μM and propidium iodide (PI; Aladdin, P113815) 1:125 to 80 μM. Prior to staining, culture medium was completely removed from both the OC‐Plex device and replaced with the staining solution. Samples were incubated in a humidified incubator (37°C, 5% CO_2_) for 2 h.

### 4.8 Image acquisition and processing

Imaging was conducted using the Molecular Devices ImageXpress Micro Confocal system with a 4× air objective. Each chip was acquired with two overlapping fields of view and 19 Z‐slices (14 μm step size) to capture the full gel channel. BF images were collected for longitudinal monitoring of cell growth and processed by stitching, projection, and background correction. For live/dead assays, Hoechst 33342 and propidium iodide were imaged in the 4′,6‐diamidino‐2‐phenylindole and tetramethylrhodamine isothiocyanate channels, respectively. Fluorescence images were stitched, background‐subtracted, and projected prior to analysis.

### 4.9 Image analyses

Image analyses were performed using the Custom Module Editor (ImageXpress Micro Confocal system). Processed BF images were utilized for cell/aggregate‐segmentation to monitor cell growth over time. Each individual cell or cluster of cells was segmented as an object based on its intensity. The changes in mean object area over time of each OC‐Plex chip were quantified to represent the average growth of individual cells or clusters. Additionally, the change in the total area of all objects over time was calculated for each chip to indicate overall cell growth at the chip level.

For viability imaging assay, the image‐analysis strategy involved defining ROI and feature extraction using a fluorescence intensity‐based masking process. Individual cells or clusters of cells were segmented as objects by applying a mask using the thresholding method based on Hoechst 33342 intensity. Within the object mask, dead cells were identified based on PI intensity. Each object was further assessed for a variety of intensity and morphology‐related metrics. The viable area was quantified by deducting the PI mask area from the Hoechst 33342 mask, and the viability% was quantified by using the viable area normalized to the sum area of all cells.

### 4.10 Cell viability assay

For viability assay, culture medium was removed from both channels of the OC‐Plex device and replaced with 100 μL CellTiter‐Glo 3D Reagent (Promega, G9681) in the medium channel and ECM ports. Devices were incubated on an orbital shaker at room temperature for 1 h. In parallel, an rATP standard (Promega, P1132) was prepared by 1:1000 dilution of a 10 mM stock in culture medium and serially diluted (50 μL transfers) across wells A1–A11 of a white opaque 96‐well plate, well A12 served as the blank. After 5 min shaking and 25 min incubation, luminescence samples from each chip were transferred to individual wells and measured immediately (0.25 s integration).

### 4.11 Statistical analyses

Data are presented as mean ± standard deviation (s.d.) unless otherwise specified in the figure legends. Graphing and statistical analyses were performed using GraphPad Prism 10.0. Comparisons between multi‐group comparisons were assessed by one‐way or two‐way ANOVA, followed by Tukey's multiple comparisons test, depending on the experimental design. *p* values <0.05 were considered statistically significant (*p* < 0.05; *p < 0.01; **p < 0.001; n.s., not significant). Dose–response curves were generated by fitting the data to a four‐parameter logistic (4PL) model in GraphPad Prism (Version 10.0). The half‐maximal inhibitory concentration (IC_50_) and AUC were calculated from the fitted curves to quantify drug potency and overall efficacy.

## AUTHOR CONTRIBUTIONS

**Hua Zhou:** Conceptualization; validation; funding acquisition; resources; investigation; writing – original draft. **Xinyu Gu:** Conceptualization; validation; resources; data curation. **Weilong Chang:** Validation; resources; investigation; data curation. **Lin Zhou:** Writing – original draft; software; visualization; data curation; formal analysis. **Ruixuan Cao:** Validation; data curation. **Xin Wang:** Validation; data curation; formal analysis. **Liuyan Deng:** Validation. **Shengping Xiao:** Formal analysis; visualization; software. **Sen Ye:** Software; formal analysis. **Xiaohua Qian:** Methodology; project administration; supervision. **Zhiyong Xie:** Software; formal analysis; visualization. **Shipeng Li:** Resources; validation; supervision. **Xin Xie:** Project administration; supervision; data curation; funding acquisition. **Haiqing Bai:** Writing – review and editing; software; project administration; supervision.

## CONFLICT OF INTEREST STATEMENT

Competing interests: Lin Zhou, Xin Wang, Ruixuan Cao, Xiaohua Qian, Zhiyong Xie, Xin Xie, Haiqing Bai are currently employed by and own equity in Xellar Inc., a company that developed an Organ Chip‐based model for drug testing and drug discovery. Other authors declare no competing interests. Correspondence and requests for materials should be addressed to Haiqing Bai.

## Supporting information


**Figure S1.** Sustained growth of patient‐derived CRC organoids in the microfluidic culture platform. (A) Representative bright‐field images showing long‐term culture of patient‐derived CRC organoids in the microfluidic chip. A large organoid with a diameter of approximately 700 μm (white arrow) is observed on day 25, demonstrating the platform's capacity to support sustained organoid growth and maturation. (B) Immunofluorescence validation of CRC organoid identity on‐chip. Bright‐field and fluorescence images confirm that the cultured organoids retain their intestinal lineage, as demonstrated by the nuclear expression of the specific marker CDX2, which co‐localizes with DAPI (white arrows). Scale bar: 200 μm.
**Figure S2.** Automated image analysis workflow for cell viability quantification. (A) A representative bright‐field (BF) image showing the defined region of interest (ROI, white outline) at the device interface. (B) Corresponding fluorescent images of the same field stained with Hoechst 33342 (nuclei, blue) and propidium iodide (PI, red). The applied segmentation masks identify all nuclei (“Single‐cell,” green mask; “Aggregate,” magenta masks) and dead cells (yellow masks). The viable area was quantified by deducting the PI mask area from Hoechst33342 mask, and the viability% was quantified by using viable area normalized to the sum area of all cells. Scale bar, 200 μm.
**Figure S3.** Quantitative comparison of drug responses assessed by imaging‐based and ATP‐based viability assays. CRC organoids were treated with irinotecan hydrochloride alone or 10 μM 5‐FU + oxaliplatin (six concentrations, 0.01–200 μM) for 4 days. Cell viability was assessed by (A) image‐based live/dead staining and (B) ATP‐based CellTiter‐Glo assay. Data are presented as mean ± SEM (*n* = 3). Two‐way ANOVA with Tukey's test; *p < 0.05, **p < 0.01, ***p < 0.001, ****p < 0.0001; ns, not significant. Irinotecan alone showed greater potency than 5‐FU + oxaliplatin across most concentrations, particularly at higher doses.
**Figure S4.** Time‐dependent drug responses assessed by imaging‐based and ATP‐based viability assays. CRC organoids were cultured for 4 days, followed by treatment with 5‐FU + oxaliplatin (FOLFOX mimic) at the indicated concentrations (0.01–200 μM). Cell viability was evaluated at 3, 4, and 6 days post‐treatment using (A) image‐based live/dead staining and (B) ATP‐based CellTiter‐Glo assay. Data are presented as mean ± SEM (*n* = 3). Statistical significance was determined by two‐way ANOVA (time × concentration), followed by Tukey's multiple comparisons test. *p < 0.05, **p < 0.01, ***p < 0.001, ****p < 0.0001; ns, not significant. A dose‐dependent reduction in viability was consistently observed across all three time points in both assay formats, and the response profiles at day 3 were highly similar to those at days 4 and 6, indicating that a 3‐day exposure is sufficient to capture the drug's efficacy trend.

**Table S1.** Organoid diameter measured at different culture time points in the microfluidic chip.
**Table S2.** Sample information.

## Data Availability

All data generated or analyzed during this study are included in this published article and its supplementary information files.
