# Supplementary material for: A patient‐derived organ‐on‐a‐chip platform for predicting chemotherapy response in locally advanced colorectal cancer
Source: Bioeng Transl Med. 2026 Nov 2:e70190. Online ahead of print. doi: 10.1002/btm2.70190 (PMC13725798; doi:10.1002/btm2.70190)
Supplement: Supplementary file 1 [file BTM2-9999-e70190-s001.docx]

**A Patient-Derived Organ-on-a-Chip Platform for Predicting** **Chemotherapy Response in Locally Advanced Colorectal Cancer**

Hua Zhou^1,2,8*^, Xinyu Gu^3,8*^, Weilong Chang ^4,8*^, Lin Zhou^2,8*^, Ruixuan Cao^2^, Xin Wang^2^, Liuyan Deng^2^, Shengping Xiao^5^, Sen Ye^5^, Xiaohua Qian^6^, Zhiyong Xie^6^, Shipeng Li^7#^, Xin Xie^2,5,6#^, Haiqing Bai^2,6#^

**Affiliations:**

^1^Endocrinology and Metabolism Center, Henan Key Laboratory of Rare Diseases, The First Affiliated Hospital, and College of Clinical Medicine of Henan University of Science and Technology, Luoyang, 471003, Henan, China.

^2^Henan Academy of Innovations in Medical Science, Zhengzhou, 450000, Henan, China.

^3^Henan Key Laboratory of Cancer Epigenetics, Cancer Institute, The First Affiliated Hospital, College of Clinical Medicine, Henan University of Science and Technology, Luoyang, 471000, Henan, China.

^4^Department of Gastrointestinal Surgery, the First Affiliated Hospital of Zhengzhou University, Zhengzhou, 450052, China.

^5^Yaosu Technology, Building 10, Caohejing Nanqiao Science and Technology Park, 6055 Jinhai Highway, Fengxian District, Shanghai 201400, China.

^6^Xellar Biosystems, Boston, MA, 02129, USA.

^7^Department of Hepatopancreaticobiliary Surgery, Henan Provincial People’s Hospital, Zhengzhou University, Zhengzhou, 450003, China.

^8^These authors contributed equally to this work (Hua Zhou, Xinyu Gu, Weilong Chang and Lin Zhou).

#Co-corresponding authors: Shipeng Li, [shipengli2010@163.com](mailto:shipengli2010@163.com); Xin Xie, [xxie@xellarbio.com](mailto:xxie@xellarbio.com); Haiqing Bai, [hbai@xellarbio.com](mailto:hbai@xellarbio.com).

**Supplementary Table 1.** **Organoid diameter measured at different culture time points in the microfluidic chip.**

| **Culture days** | **Organoid diameter (mean ± SD, μm)** |
| --- | --- |
| Day 1 | 25.1 ± 1.7 |
| Day 3 | 59.0 ± 3.7 |
| Day 5 | 100.6 ± 4.7 |
| Day 7 | 141.7 ± 5.4 |
| Day 9 | 207.2 ± 5.9 |

**Supplementary Table 2. Sample information.**

| **Patient ID** | **Gender** | **Age** | **BMI** | **Tumor Site** | **Tumor Grading** | **Treatment before surgery** | **Treatment after surgery** |
| --- | --- | --- | --- | --- | --- | --- | --- |
| 001 | Female | 54 | 22.31 | colon | T2N0M0 | None | None |
| 005 | Male | 67 | 23.14 | rectum | T3N0M0 | None | XELOX |
| 007 | Male | 57 | 28.41 | colon | T3N0M0 | None | mFOLFOX6 |
| 009 | Male | 57 | 26.12 | rectum | T3N0M0 | None | FOLFOX |
| 010 | Female | 68 | 21.72 | rectum | T3N0M0 | None | mFOLFOX6 |
| 011 | Female | 58 | 24.97 | rectum | T3N0M0 | None | mFOLFOX6 |
| 014 | Female | 79 | 27.33 | rectum | T3N0M0 | None | None |
| 015 | Female | 66 | 24.24 | colon | T3N2aM0 | None | FOLFOX6 |
