# Supplementary material for: A patient‐derived organ‐on‐a‐chip platform for predicting chemotherapy response in locally advanced colorectal cancer
Source: Bioeng Transl Med. 2026 Nov 2:e70190. Online ahead of print. doi: 10.1002/btm2.70190 (PMC13725798; doi:10.1002/btm2.70190)
Supplement: Supplementary file 2 [file BTM2-9999-e70190-s002.docx]

**A Patient-Derived Organ-on-a-Chip Platform for Predicting** **Chemotherapy Response in Locally Advanced Colorectal Cancer**

Hua Zhou^1,2,8*^, Xinyu Gu^3,8*^, Weilong Chang ^4,8*^, Lin Zhou^2,8*^, Ruixuan Cao^2^, Xin Wang^2^, Liuyan Deng^2^, Shengping Xiao^5^, Sen Ye^5^, Xiaohua Qian^6^, Zhiyong Xie^6^, Shipeng Li^7#^, Xin Xie^2,5,6#^, Haiqing Bai^2,6#^

**Affiliations:**

^1^Endocrinology and Metabolism Center, Henan Key Laboratory of Rare Diseases, The First Affiliated Hospital, and College of Clinical Medicine of Henan University of Science and Technology, Luoyang, 471003, Henan, China.

^2^Henan Academy of Innovations in Medical Science, Zhengzhou, 450000, Henan, China.

^3^Henan Key Laboratory of Cancer Epigenetics, Cancer Institute, The First Affiliated Hospital, College of Clinical Medicine, Henan University of Science and Technology, Luoyang, 471000, Henan, China.

^4^Department of Gastrointestinal Surgery, the First Affiliated Hospital of Zhengzhou University, Zhengzhou, 450052, China.

^5^Yaosu Technology, Building 10, Caohejing Nanqiao Science and Technology Park, 6055 Jinhai Highway, Fengxian District, Shanghai 201400, China.

^6^Xellar Biosystems, Boston, MA, 02129, USA.

^7^Department of Hepatopancreaticobiliary Surgery, Henan Provincial People’s Hospital, Zhengzhou University, Zhengzhou, 450003, China.

^8^These authors contributed equally to this work (Hua Zhou, Xinyu Gu, Weilong Chang and Lin Zhou).

#Co-corresponding authors: Shipeng Li, [shipengli2010@163.com](mailto:shipengli2010@163.com); Xin Xie, [xxie@xellarbio.com](mailto:xxie@xellarbio.com); Haiqing Bai, [hbai@xellarbio.com](mailto:hbai@xellarbio.com).

**Supplementary Figure 1. Sustained growth of patient-derived CRC organoids in the microfluidic culture platform.** (A) Representative bright‑field images showing long‑term culture of patient‑derived CRC organoids in the microfluidic chip. A large organoid with a diameter of approximately 700 μm (white arrow) is observed on day 25, demonstrating the platform's capacity to support sustained organoid growth and maturation. (B) Immunofluorescence validation of CRC organoid identity on‑chip. Bright‑field and fluorescence images confirm that the cultured organoids retain their intestinal lineage, as demonstrated by the nuclear expression of the specific marker CDX2, which co‑localizes with DAPI (white arrows). Scale bar: 200 μm.

**Supplementary Figure 2. Automated image analysis workflow for cell viability quantification**. (**A**) A representative bright-field (BF) image showing the defined region of interest (ROI, white outline) at the device interface. (**B**) Corresponding fluorescent images of the same field stained with Hoechst 33342 (nuclei, blue) and propidium iodide (PI, red). The applied segmentation masks identify all nuclei (“Single-cell”, green mask; “Aggregate”, magenta masks) and dead cells (yellow masks). The viable area was quantified by deducting the PI mask area from Hoechst33342 mask, and the viability% was quantified by using viable area normalized to the sum area of all cells. Scale bar, 200 μm.

**Supplementary Figure 3. Quantitative comparison of drug responses assessed by imaging‑based and ATP‑based viability assays**. CRC organoids were treated with irinotecan hydrochloride alone or 10 μM 5‑FU + oxaliplatin (six concentrations, 0.01–200 μM) for 4 days. Cell viability was assessed by **(A)** image‑based live/dead staining and **(B)** ATP‑based CellTiter‑Glo assay. Data are presented as mean ± SEM (n = 3). Two‑way ANOVA with Tukey's test; *p < 0.05, **p < 0.01, ***p < 0.001, ****p < 0.0001; ns, not significant. Irinotecan alone showed greater potency than 5‑FU + oxaliplatin across most concentrations, particularly at higher doses.

**Supplementary Figure 4. Time‑dependent drug responses assessed by imaging‑based and ATP‑based viability assays**. CRC organoids were cultured for 4 days, followed by treatment with 5‑FU + oxaliplatin (FOLFOX mimic) at the indicated concentrations (0.01–200 μM). Cell viability was evaluated at 3, 4, and 6 days post‑treatment using **(A)** image‑based live/dead staining and **(B)** ATP‑based CellTiter‑Glo assay. Data are presented as mean ± SEM (n = 3). Statistical significance was determined by two‑way ANOVA (time × concentration), followed by Tukey's multiple comparisons test. *p < 0.05, **p < 0.01, ***p < 0.001, ****p < 0.0001; ns, not significant. A dose‑dependent reduction in viability was consistently observed across all three time points in both assay formats, and the response profiles at day 3 were highly similar to those at days 4 and 6, indicating that a 3‑day exposure is sufficient to capture the drug's efficacy trend.
